# Production of microbial transglutaminase by *Streptoverticillium cinnamoneum* KKP 1658

**DOI:** 10.17179/excli2024-7033

**Published:** 2024-05-03

**Authors:** Vitaliy Kolotylo, Kamil Piwowarek, Marek Kieliszek

**Affiliations:** 1 Department of Food Biotechnology and Microbiology, Institute of Food Sciences, Warsaw University of Life Sciences-SGGW, Nowoursynowska 159 C, 02-776 Warsaw, Poland

**Keywords:** microbial transglutaminase, Streptoverticillium cinnamoneum, biosynthesis, waste products

## Abstract

Transglutaminase finds broad applications in the food industry, influencing texture, shelf life and overall food quality. It can be utilized to create products with enhanced sensory and technological properties and serves as a tool to reduce food waste. The aim of this study was to optimize the production of microbial transglutaminase (MTG) by the genetically unmodified strain of *Streptoverticillium cinnamoneum* KKP 1658. Tryptone soy broth (TSB) was chosen as the optimal inoculation medium due to its high MTG activity in the cultivation substrate. The optimal inoculum incubation time was determined as 24 hours, with a dosage of 10 %. Various nitrogen sources were investigated while maintaining a consistent nitrogen dosage (0.2 %) (including aminobak, corn steep liquor, ammonium nitrate and ammonium sulphate) to achieve the highest microbiological transglutaminase activity. The combination of aminobak with corn steep liquor and a cultivation period of 72 hours (28 °C; pH 6.0-6.5) yielded the highest MTG activity at 6.59 U/mL.

## Introduction

Genetically modified microorganisms (GMMs) have found applications across various industries, including agriculture, pharmaceuticals, environmental management (in pollution control contexts) and in sectors such as food, paper and textiles manufacturing (Mallikarjuna and Yellamma, 2019[20]). Enzymes in the food industry are increasingly gaining significance (Raveendran et al., 2018[24]), with particular attention directed towards microbial transglutaminase (MTG) produced by the genetically unmodified strain of streptomycetes from the genus *Streptoverticillium*. Transglutaminase enzyme (E.C. 2.3.2.13) is currently one of the most widely utilized enzymes in protein cross-linking processes (Lerner et al., 2023[18]). MTG stimulates the formation of both intra- and intermolecular cross-links by creating durable, covalent bonds between lysine amino acids and glutamine residues in proteins (Ceresino et al., 2018[3]).

It is worth noting that despite the numerous advantages of genetic engineering in MTG production (enhanced activity, thermostability, and efficiency) (Juettner et al., 2018[14]), there are still certain drawbacks and challenges. A segment of society opposes genetic engineering processes (some countries impose bans or strict restrictions) and consistently expresses resistance, partly due to ethical concerns, unexplored long-term consequences, beliefs in the induction of diseases, carcinogenicity and allergies to GMMs or their metabolites (Dzhumanova and Nazarova 2022[5]). Additional limitations in GMM production include labor-intensive and costly research, the creation of mutants on a large scale and unforeseen side effects (Akbari et al., 2021[1]).

Producing food products involving GMMs raises questions about their safety for human health and the environment. GMMs may pose risks; therefore, it is crucial to provide adequate data when conducting risk assessments associated with them (EUR-Lex 2009[8]; EFSA, 2011[6]). Newly expressed proteins from GMMs are assessed for similarity to allergenic proteins and are also tested for horizontal gene transfer (antibiotic resistance markers). The transfer of antibiotic resistance to other microorganisms can threaten the effectiveness of antibiotics. GMMs may have the ability to colonize the human gastrointestinal tract, potentially affecting the normal microbiome (a collection of microorganisms, mainly bacteria, in the human digestive tract that plays a crucial role in digestion, metabolism, immunity and overall health) (Dzhumanova and Nazarova, 2022[5]; Wesseler et al., 2023[28]).

Owing to the ongoing concerns surrounding GMMs in certain circles, it is imperative to explore alternative solutions, particularly in the food industry, without the use of genetically modified organisms and their metabolites. Microbial transglutaminase represents a significant area of interest in the food industry due to its practical benefits (such as improving food quality, expanding product ranges and increasing product shelf life) and economic advantages (e.g., reducing production costs, minimizing waste and decreasing the need for chemical additives). On an industrial scale, transglutaminase is utilized in the baking, meat, fish, dairy and even pharmaceutical industries. Many scientists recommend the use of MTG in various food products, including low-fat and low-salt meat products, fish products (fish paste), cheeses (tofu, cottage cheese and aged cheeses), yogurts, breads and pasta (Kolotylo et al., 2023[17]). Commercial transglutaminase is produced by *Streptoverticillium mobaraensis *on an industrial scale, but this system has certain drawbacks, including low enzyme efficiency and high costs associated with nutrient media (Akbari et al., 2021[1]).

In the current study, an attempt was made to obtain natural production of MTG by the strain *Streptoverticillium cinnamoneum* KKP 1658 by selecting appropriate production parameters to achieve the best possible MTG activity while maintaining the economic benefits associated with expensive components of research substrates (e.g., aminobak, peptone, yeast extract (Kieliszek and Misiewicz, 2014[15]; Akbari et al., 2021[1]). The emphasis of this work is on using a genetically unmodified strain and naturally enhancing MTG activity with the goal of potential industrial-scale utilization, offering advantages such as food safety (no risk of unpredictable side effects), consumer acceptance and trust, cost reduction and circumventing GMO regulations in countries with stringent rules, facilitating the production process and the introduction of new products to the market.

## Methodology

### Biological material

The research utilized the streptomycete strain *Streptoverticillium cinnamoneum* KKP 1658 obtained from the Culture Collection of the Department of Biotechnology and Food Microbiology, Warsaw University of Life Sciences. The strain was stored at -80 °C in Tryptone Soy Broth (TSB) liquid medium with glycerol.

### Cultivation methods

Inoculation medium used for the study was TSB. TSB composition [g/L]: casein peptone 17; soy peptone 3; NaCl 5; K_2_HPO_4_ 2.5; glucose 2.5. An additional inoculation medium (O_3_) consisted of [g/L]: oat flakes 25, aminobak 2, K_2_HPO_4_ 2, and MgSO_4_*7H_2_O 1. The initial pH of the media was adjusted to 7.0 (pH meter Elmetron CP-505, Poland). Media were sterilized at 121 °C for 15 minutes (Autoclave Systec D-45, De Ville, Poland).

The inoculation medium was inoculated with the *Streptoverticillium cinnamoneum* KKP 1658 strain (6-8×10^7^ CFU/mL) and placed on a shaker (Eppendorf Innova 44 Incubator Shaker, Germany) for cultivation at 28 °C, with rotary shaking at 180 revolutions per minute for 72 hours for adaptation and biomass multiplication. After 72 hours, the inoculum was transferred to the research medium (the inoculum constituted 10 % of the medium volume), and cultivations were conducted at four time points (24, 48, 72 and 96 hours) at 28 °C. Composition of the research medium [g/L]: soluble starch 20, aminobak 20, yeast extract 2, KH_2_PO_4_ 2, Na_2_HPO_4_ 2, MgSO_4_*7H_2_O 1. The pH of the medium was adjusted to 6.5 and sterilised at 121 °C for 15 minutes (Autoclave Systec D-45, De Ville, Poland). Cultivations were conducted in triplicates.

To determine the appropriate nitrogen source in subsequent research media, aminobak was replaced with: ammonium sulphate (NH_4_)_2_SO_4_), ammonium nitrate (NH_4_NO_3_), corn steep liquor and aminobak with corn steep liquor. The nitrogen sources were applied separately, maintaining the nitrogen content in the research media at the same level (0.2 %).

### Biomass yield determination

The biomass yield of the *S. cinnamoneum* KKP 1658 strain was measured after centrifugation (Eppendorf 5810 Centrifuge, Germany) (4500 rpm; 10 min) of 30 mL of post-culture fluid in pre-weighed and dried 50 mL Falcon tubes. The supernatant was decanted for transglutaminase activity determination, and the sediment was dried (SML Zalmed Dryer, Poland) (80 °C) to a constant mass. The biomass yield after centrifugation and drying was calculated per 1 L of medium and expressed in grams of dry substance (g_d.w._/L) in three repetitions.

### Transglutaminase activity determination

**Figure 1:**
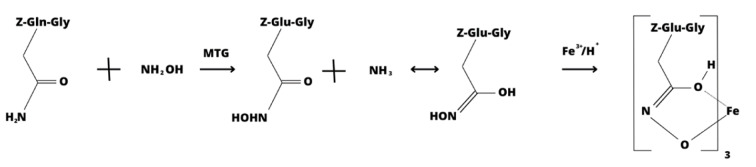
Chemical reaction series illustrating the principle of the enzymatic test for detecting MTG.

**Figure 2:**
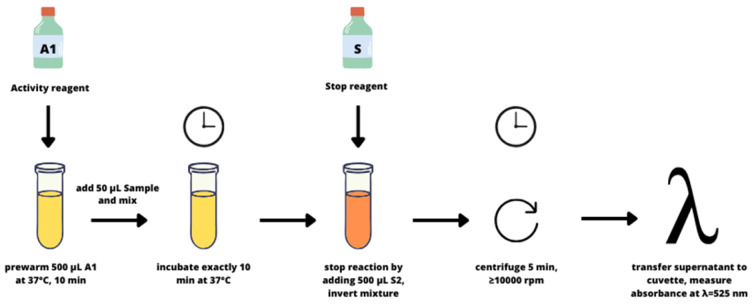
Schematic representation of the procedure for performing the MTG assay.

The enzymatic activity of transglutaminase was determined using commercial tests, Microbial Transglutaminase Assay Kit Art. No. Z009 (Zedira GmbH, Darmstadt, Germany). The kit for microbial transglutaminase determination utilizes the substrate Z-Gln-Gly (N2-[(phenylmethoxy)carbonyl]-L-glutaminyl-glycine, C_15_H_19_N_3_O_6_) as the amine acceptor and hydroxylamine as the amine donor. In the presence of MTG, hydroxylamine is incorporated into the Z-Gln-Gly substrate, forming Z-glutamyl-hydroxamic-glycine, which forms a colorful complex with iron (III) detectable spectrophotometrically (BIO-RAD SmartSpec 3000 Spectrophotometer, Poland) at a wavelength of 525 nm (Figure 1(Fig. 1) and 2(Fig. 2)). 

One unit of transglutaminase activity (U) is defined as the amount of enzyme that catalyzes the formation of 1 µmole of hydroxamic acid per minute in the reaction between Z-Gln-Gly and hydroxylamine at pH 6.0 and a temperature of 37 °C (Folk and Cole, 1966[11]).

### Evaluation of inoculum material impact

The study investigated how the pre-cultivation process in the inoculation medium (O_3_) with a different composition (subsection, as described earlier) than the TSB affects the biomass yield and the activity of transglutaminase produced by the examined strain of *Streptoverticillium cinnamoneum* KKP 1658 in the research medium with aminobak and corn steep liquor.

To assess the influence of the age of the inoculum material on the biomass yield and the activity of the produced transglutaminase, pre-cultivation of *S. cinnamoneum* KKP 1658 was carried out for 24, 48 and 72 hours, followed by a 96-hour research cultivation. The effect of the inoculum dose on MTG activity was examined based on results obtained for 5, 10 and 15 % inoculum doses relative to the volume of the research medium.

### Assessment of initial pH influence on medium

Changes in pH values during the 96-hour cultivation of *S. cinnamoneum* KKP 1658 in the experimental medium with the addition of aminobak and corn steep liquor, inoculated with a 24-hour inoculum at a dose of 10 %, were examined. The initial pH levels of 5.0, 5.5, 6.0, 6.5 and 7.0 were investigated to determine their impact on the activity of the produced transglutaminase.

### Statistical analysis

The obtained research results underwent analysis of variance using the Statistica 13.3 software. All analyses were performed in triplicate. Significance differences between means in individual groups were verified using Tukey's HSD test at a significance level of α = 0.05. The normal distribution was confirmed by conducting the Shapiro-Wilk test.

## Results

### A biomass yield of S. cinnamoneum KKP 1658 in media with different nitrogen sources

**Figure 3:**
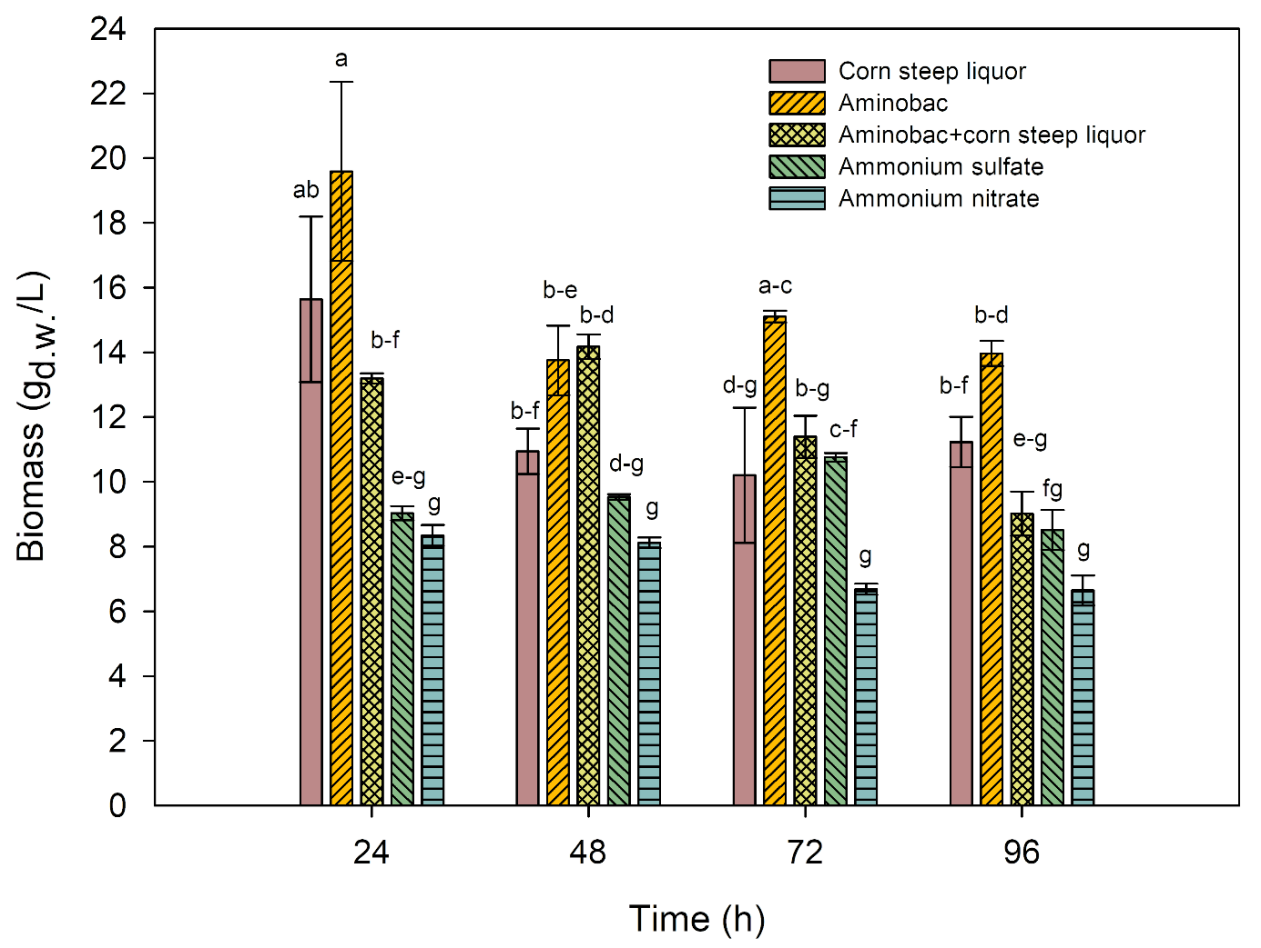
Biomass yield for *S. cinnamoneum* strain with various nitrogen sources in a 96-hour cultivation. ^a-g^ Means with the same letter did not differ significantly.

Determining the biomass yield provides insight into the relationship between this parameter and the activity of the obtained transglutaminase. This is a crucial aspect because an increased biomass yield does not always translate to higher enzyme activity. The impact of various nitrogen sources on the biomass yield of the examined strain of *Streptoverticillium cinnamoneum *KKP 1658 was investigated. A commercial nitrogen source (aminobak), a waste nitrogen source (corn steep liquor), partial substitution of aminobak with corn steep liquor and inorganic nitrogen sources in the form of ammonium salts - sulphate and nitrate (Figure 3(Fig. 3)) - were utilized in the study. The medium with commercial aminobak was treated as the control sample. Additionally, a cultivation was conducted in the research medium without aminobak, yielding stabilized biomass results of 7.6, 7.9, 7.7 and 7.4 g_d.w_./L after 24, 48, 72 and 96 hours of cultivation, respectively.

The highest biomass yield reaching 19.59 g _d.w._/L was obtained after a 24-hour cultivation period in a medium supplemented with aminobak. Similarly statistically significant results were recorded for a medium containing waste corn steep liquor, yielding 15.64 g _d.w._/L in the same time. The combination of aminobak and corn steep liquor resulted in a biomass yield of 13.19 g _d.w._/L after 24 hours, significantly different from the aminobak-only medium but statistically similar to the corn steep liquor medium. Biomass yields from media with inorganic nitrogen salts were the lowest among the tested substrates, measuring 9.03 and 8.34 g _d.w._/L for ammonium sulphate and ammonium nitrate, respectively. These results did not differ statistically. The biomass yield from media with inorganic nitrogen sources was only 9.77 % higher than the nitrogen-free medium (7.6 g _d.w._/L after 24 hours) for ammonium nitrate, and 18.8 % higher for ammonium sulphate.

On the second day of cultivation, changes in biomass yield results were observed. The highest biomass yield (14.17 g _d.w._/L) was recorded for the medium combining aminobak and corn steep liquor as the primary nitrogen source. Biomass yields in four out of five tested media did not differ statistically. The results for media with aminobak and corn steep liquor alone were 13.75 and 10.94 g _d.w._/L, respectively. After 48 hours of cultivation, the biomass yield in the ammonium sulphate medium (9.53 g _d.w._/L) did not differ statistically from the biomass obtained in the corn steep liquor medium. The lowest yield after 48 hours of cultivation was observed in the ammonium nitrate medium (8.12 g _d.w._/L), which was only 2.6 % higher than the biomass from the nitrogen-free medium (7.9 g _d.w._/L).

In summary, the highest biomass yields were obtained for the medium with commercial aminobak. Substrates with corn steep liquor and a combination of aminobak with corn steep liquor exhibited slightly lower results, but these differences were not statistically significant. Substrates with inorganic nitrogen forms showed the lowest biomass yields compared to the others. It is noteworthy that ammonium nitrate consistently resulted in the lowest biomass yield in each time unit, often lower than the nitrogen-free medium after 72 and 96 hours of cultivation.

### MTG activity of S. cinnamoneum KKP 1658

**Figure 4:**
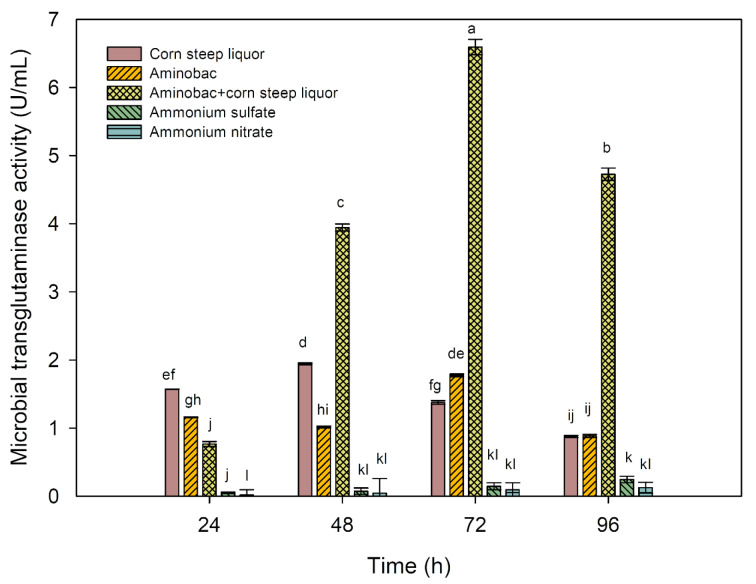
MTG activity produced by *S. cinnamoneum s*train with different nitrogen sources in a 96-hour culture. ^a-l^ Means with the same letter did not differ significantly.

The culture supernatant underwent enzymatic assays to determine microbiological transglutaminase (MTG) activity. Significant differences in MTG activity were observed depending on the nitrogen source used in cultivation (Figure 4(Fig. 4)). Like the biomass, substrates with inorganic nitrogen salts exhibited the lowest activity of the assayed enzyme. After 96 hours of cultivation, the highest MTG activity for ammonium sulphate as the nitrogen source was only 0.24 U/mL, representing the highest MTG activity for ammonium salts obtained in this study.

After 24 hours of cultivation, the highest transglutaminase activity was observed in the medium with corn steep liquor, reaching 1.57 U/mL. Amino peptone resulted in a significantly lower activity of 1.16 U/mL. The combination of amino peptone with corn steep liquor after 24 hours of cultivation led to an MTG activity of 0.77 U/mL. In the next 24 hours (48 h), the transglutaminase activity for the corn steep liquor medium increased significantly to 1.94 U/mL, while the MTG activity in the amino peptone medium stabilized at 1 U/mL. After 48 hours of cultivation, a substantial increase in MTG activity was observed for the medium with amino peptone and corn steep liquor, reaching 3.94 U/mL. It is noteworthy that after the next 24 hours of cultivation (72 h), the transglutaminase activity reached 6.59 U/mL and decreased to 4.73 U/mL on the last day (96 h). For the media with amino peptone and corn steep liquor alone, it was not possible to achieve transglutaminase activity exceeding 2 U/mL throughout the entire cultivation period. Referring to the determination of biomass yield, it can be presumed that the highest biomass yield does not always translate into obtaining the highest activity of the secreted enzyme.

At this stage of the research, it was found that by choosing the experimental medium with amino peptone and corn steep liquor, the highest transglutaminase activity in the culture broth can be obtained by cultivating it for 72 hours. The next stages of the study aimed to examine the impact of inoculum material (type, age and dose) and the initial pH of the medium on transglutaminase activity. The results of these studies are presented in the following subsections.

### The influence of inoculum material on biomass yield and MTG biosynthesis

The medium used for pre-culturing actinomycetes plays a crucial role in optimizing transglutaminase production (Kieliszek and Misiewicz, 2014[15]). The selection of a suitable inoculation medium, its optimal dosage and its pre-culturing time are essential for obtaining appropriate starter inoculum for subsequent MTG production.

**Figure 5:**
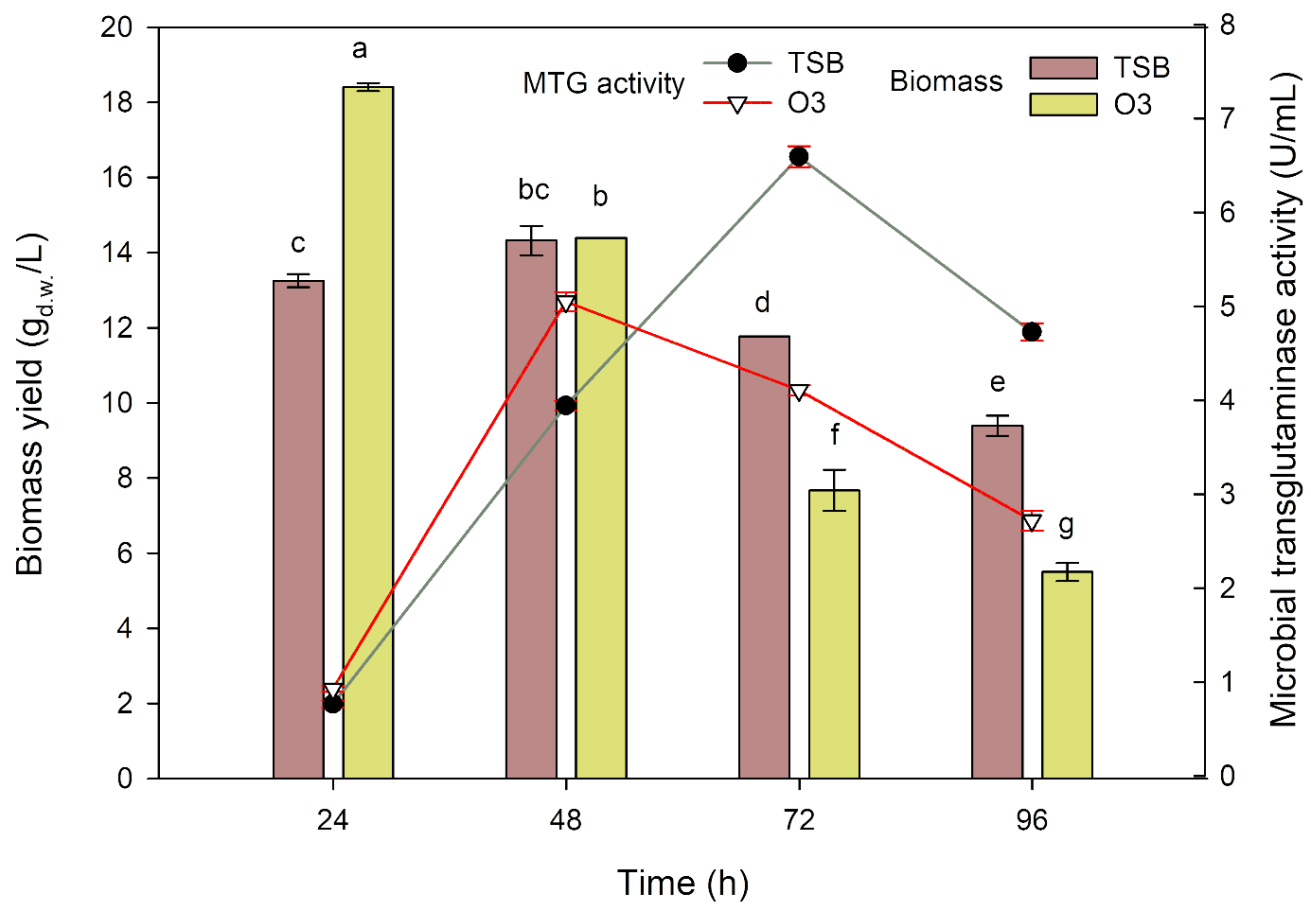
Changes in biomass yield and MTG production depending on the applied inoculation medium using aminobak/corn steep liquor 1:1 as the main nitrogen source in a 96-hour cultivation. ^a-g^ Means with the same letter did not differ significantly.

The impact of pre-culturing in the inoculation medium (O_3_), which had a different composition than the TSB medium, on biomass yield and the activity of the obtained transglutaminase (Figure 5(Fig. 5)) for the investigated strain *Streptoverticillium cinnamoneum* KKP 1658 in the experimental medium with amino peptone and corn steep liquor (as main nitrogen sources) was examined.

After a 24-hour cultivation using the O_3_ inoculation medium, the highest biomass yield of *Streptoverticillium cinnamoneum* KKP 1658 was obtained, reaching 18.4 g _d.w._/L. In the case of using the TSB medium, the biomass yield result was significantly lower compared to the O_3_ medium, amounting to 13.25 g _d.w._/L. After 48 hours of cultivation, comparable biomass yield results were obtained for both tested inoculation media - 14.32 and 14.39 g _d.w._/L for TSB and O_3_, respectively. On the third day of cultivation, the biomass yield significantly decreased for both cases to 11.77 and 7.67 g _d.w._/L, respectively, for TSB and O_3_. In the last day of cultivation, even lower biomass yield results were recorded - 9.39 g _d.w._/L for TSB and 5.5 g _d.w._/L for the medium with oat flakes. A proportional relationship for biomass yield results using the O_3_ inoculation medium can be observed - increasing cultivation time leads to a decrease in the yield of the investigated strain. For the TSB inoculation medium, the maximum biomass increase was noted after 48 hours of cultivation, which, as with O_3_, decreased over the course of cultivation. The obtained values suggest that after pre-cultivation in the medium with oat flakes, the investigated strain reaches the log phase more quickly. It can be speculated that the pre-cultivation process in the TSB medium translates into an extension of the log growth phase (prolonged trophophase) in *S. cinnamoneum* KKP 1658; hence, the idiophase is delayed compared to the inoculation medium with oat flakes.

Undoubtedly, transglutaminase production is related to cell growth (Zheng et al., 2002[33]). However, an important aspect in this study was to examine how each changing parameter (inoculation medium, its dose and age; nitrogen source or cultivation parameters) affects the activity of the secreted trans-glutaminase. Regardless of the inoculation medium used, the results of MTG activity after 24 hours of cultivation did not exceed 1 U/mL (0.77 and 0.93 U/mL for TSB and O_3_, respectively) and did not differ statistically. In the next day of cultivation, the results showed a significant increase, reaching values of 3.94 and 5.05 U/mL, respectively, for the TSB and O_3_ inoculation media. After 72 hours of cultivation, the highest transglutaminase activity was recorded, obtained from the TSB medium for biomass pre-cultivation (6.59 U/mL). Pre-cultivating biomass in the medium with oat flakes for 72 hours allowed achieving MTG activity at the level of 4.1 U/mL, which was significantly lower compared to the same inoculation medium from the previous day and compared to the TSB medium from the same time unit. The results obtained after 96 hours of cultivation were at the level of 4.73 and 2.72 U/mL, respectively, for the TSB and O_3_ inoculation media.

The obtained results indicate that the use of TSB allows significantly higher transglutaminase activity (6.59 U/mL) than the O_3_ medium with oat flakes (5.05 U/mL). It is noteworthy that the maximum MTG production took 72 hours for biomass pre-cultivated in the TSB medium, and 48 hours for the O_3_ medium. This observation aligns with previous conclusions that cells of *S. cinnamoneum* KKP 1658 exhibit an extended initial growth phase in the experimental medium after pre-cultivation in the TSB medium compared to the O_3_ medium. The use of the O_3_ medium results in a faster adaptation to the environment and the initiation of transglutaminase production.

### Impact of inoculum age on MTG activity

**Figure 6:**
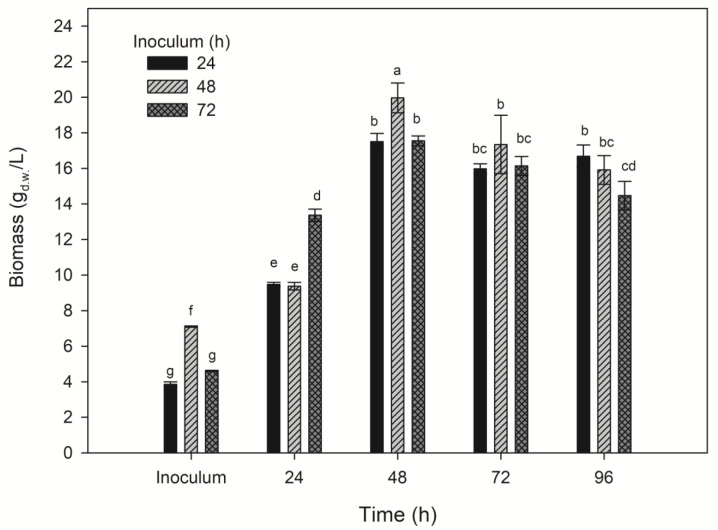
The impact of pre-cultivating biomass of *S. cinnamoneum* for 24, 48, and 72 hours on the biomass yield in a 96-hour cultivation. ^a-g^ Means with the same letter did not differ significantly.

**Figure 7:**
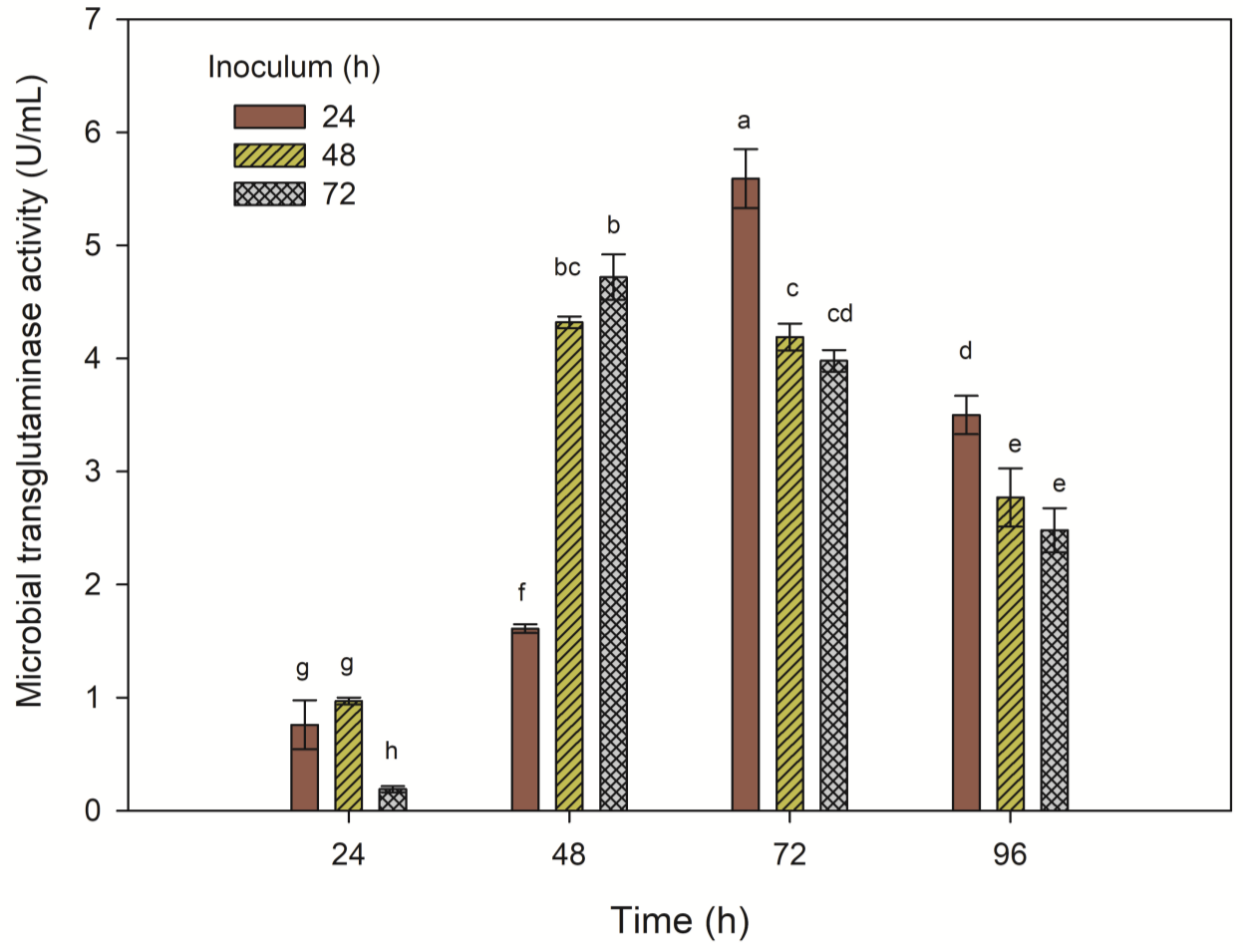
The influence of pre-cultivating biomass of *S. cinnamoneum* for 24, 48, and 72 hours on the activity of MTG in a 96-hour cultivation. ^a-h^ Means with the same letter did not differ significantly.

Considering the above studies, the TSB inoculation medium remained optimal despite the need for a 72-hour cultivation. Additional studies were conducted to determine the optimal pre-cultivation time of cells in the TSB inoculation medium. The effect of pre-cultivation for 24, 48 and 72 hours on biomass yield (Figure 6(Fig. 6)) and the activity of the obtained transglutaminase (Figure 7(Fig. 7)) was examined. The biomass yield in the inoculation medium was measured before inoculating the experimental media. Pre-cultivating for 48 hours in the TSB medium resulted in a higher biomass yield (7.1 g _d.w._/L) compared to 24 hours (3.86 g _d.w._/L) and 72 hours (4.61 g _d.w._/L).

After 24 hours of proper cultivation, the highest biomass yield was observed in the medium inoculated with a 72-hour inoculum - 13.37 g _d.w_./L. Media inoculated with 24 and 48-hour inocula showed significantly lower biomass yields (9.37-9.47 g _d.w._/L). The highest biomass yield was obtained after 48 hours of cultivation in the medium supplemented with a 48-hour inoculum (19.97 g _d.w._/L), and the biomass from the other media exhibited significantly lower yields in both this and subsequent time units. Most of the other results characterized biomass yields ranging from 15.91 to 17.34 g _d.w._/L.

After 24 hours of cultivation of the *S. cinnamoneum *KKP 1658 strain in a cultivation medium with aminobak and corn steep liquor, regardless of the age of the inoculum used, the activity of the measured transglutaminase did not exceed 1 U/mL. A significant increase was observed in the next day of cultivation, with media inoculated with 48- and 72-hour inocula showing transglutaminase activities of 4.32 and 4.72 U/mL, respectively. The highest biomass increment did not translate into the highest MTG activity, as the highest result was obtained for the medium supplemented with a 24-hour inoculum and proper cultivation for 72 hours (5.59 U/mL). Transglutaminase activity in other media on the third day of cultivation was significantly lower, as well as on the last day of cultivation.

The conducted studies provided essential information regarding the optimal time for pre-cultivation of the investigated strain of streptomycetes. It was established that the highest results of transglutaminase activity are achieved by conducting the cultivation of the *S. cinnamoneum* KKP 1658 strain for 72 hours and the pre-cultivation process in the TSB inoculation medium for 24 hours.

### The impact of different inoculum doses on MTG activity

**Figure 8:**
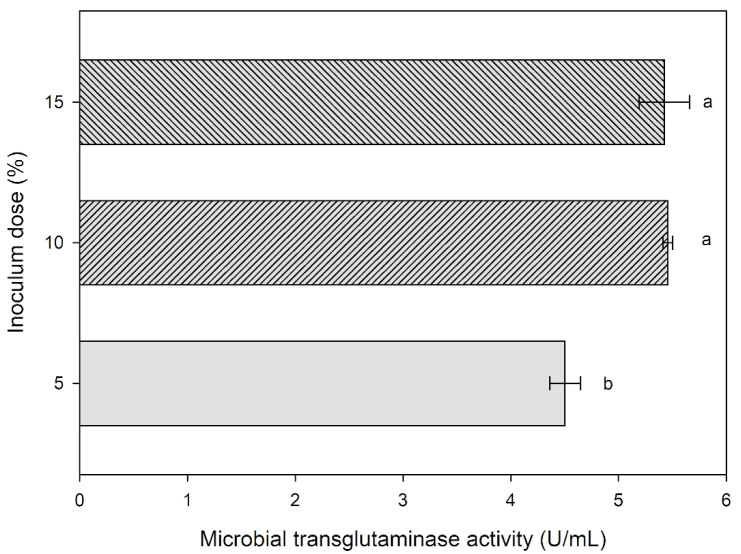
The influence of inoculum dose in the research medium on the activity of transglutaminase produced by *S. cinnamoneum.*^ a-b^ Means with the same letter did not differ significantly.

The next stage of the research aimed to examine how varying inoculum doses affect transglutaminase activity. For this purpose, inoculum in quantities of 5, 10 and 15 % was added to the research medium with aminobak and corn steep liquor. Subsequently, cultivations were conducted for 72 hours, after which the activity of the produced transglutaminase was examined. The results are presented in Figure 8(Fig. 8).

The addition of an inoculum suspension in a quantity of 5 % to the research medium resulted in transglutaminase activity of 4.5 U/mL after 72 hours of cultivation. The 5 % inoculum dose significantly decreased the MTG activity compared to the medium with a 10 % inoculum, which reached 5.46 U/mL. It was observed that an increased inoculum dose of 15 %, in comparison to the 10 % dose, did not have a statistically significant effect on the activity of the obtained transglutaminase (5.43 U/mL).

In summary, a 5 % inoculum dose significantly reduced MTG activity, while a 15 % dose did not have a statistically significant impact on MTG activity compared to the mean 10 % dose. Adding a 10 % inoculum suspension to the research medium and conducting cultivation for 72 hours ensures the highest efficiency in the production of transglutaminase by the investigated strain *Streptoverticillium cinnamoneum* KKP 1658.

### Impact of initial medium pH on MTG biosynthesis

The rate of MTG production, its activity, and efficiency in research media vary depending on the medium composition and environmental conditions, including pH. To achieve the maximum speed of MTG production, it is necessary to determine the optimal conditions for cell growth and MTG production during fermentation. The influence of pH on MTG production is a significant but underexplored aspect (Meiying et al., 2002[21]).

**Figure 9:**
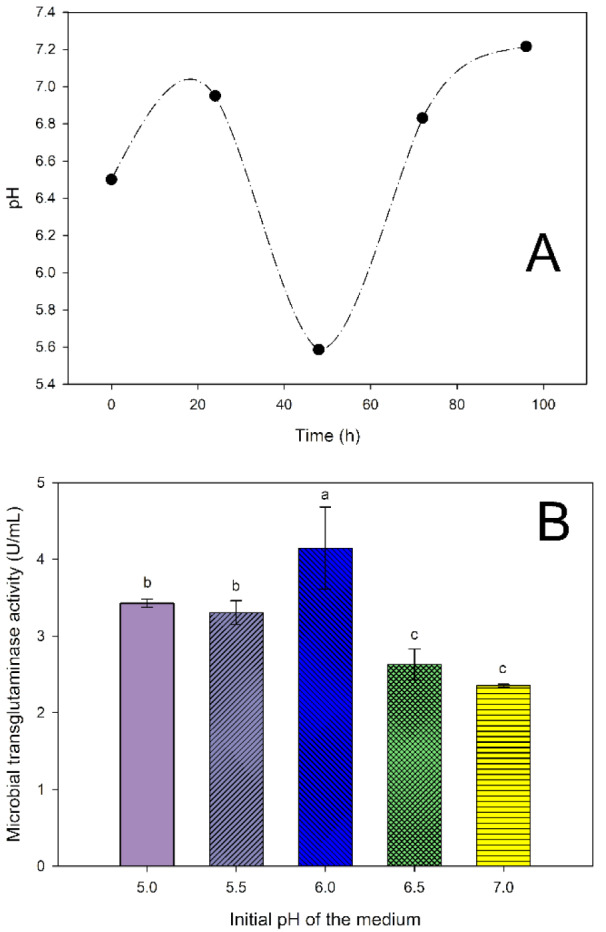
pH changes during a 96-hour cultivation of *S. cinnamoneum* with an initial pH of 6.5 (A) and the influence of initial pH on MTG activity (B). ^a-c^ Means with the same letter did not differ significantly.

Based on the available literature, the initial pH of the experimental substrate was set at 6.5, while the initial pH of the inoculation medium was set at 7.0 (Meiying et al., 2002[21]; Kieliszek and Misiewicz, 2014[15]). Changes in pH values were examined during the 96-hour cultivation of *S. cinnamoneum* KKP 1658 in the experimental medium with the addition of aminobak and corn steep liquor, inoculated with a 24-hour inoculum at a 10 % dose, confirming the optimal dose results (Figure 9A(Fig. 9)). In the first day of cultivation, an increase in pH was observed, reaching 6.95, which decreased to 5.59 after 48 hours of cultivation. After 72 hours of cultivation, where previous studies noted the highest values of MTG activity, the pH increased to 6.83, and in the last day of cultivation, it rose to 7.22.

Figure 9A(Fig. 9) depicts pH changes ranging from 5.5 to 7.25, so the influence of initial pH levels from 5.0 to 7.0 on the activity of the obtained transglutaminase was examined (Figure 9B(Fig. 9)). The highest transglutaminase activity was achieved for the substrate with an initial pH of 6.0 - 4.14 U/mL. Another homogeneous group consisted of substrates with initial pH levels of 5.0 and 5.5, which exhibited MTG activity of 3.43 and 3.31 U/mL, respectively. The lowest activity of microbial transglutaminase was observed in substrates with initial pH levels of 6.5 and 7.0: 2.63 and 2.35 U/mL, respectively.

## Discussion

Microbial transglutaminase is produced by various bacteria, fungi and actinomycetes. Numerous studies have been conducted to identify microorganisms capable of producing this enzyme (Kieliszek and Misiewicz, 2014[15]; Akbari et al., 2021[1]). These investigations have revealed that strains of *Streptomyces* sp. CBMAI 1617 (SB6) (Ceresino et al., 2018[3]) and *Actinomycetes* (Eshra et al., 2015[7]) exhibit the highest (~6 U/mL) and lowest (~0.04 U/mL) activity of this discussed enzyme, respectively.

The discovery and isolation of the *Streptomyces mobaraense* strain (Washizu et al., 1994[27]) represented the first step toward its widespread commercial use. Subsequently, many different strains of microorganisms, such as *Streptomyces lydicus* (Færgemand and Qvist, 1997[9]), *Streptomyces cinnamoneum* CBS 683.68 (Duran et al., 1998[4]) and *Streptomyces* sp. CBMAI 837 (Macedo et al., 2007[19]), were identified to produce transglutaminase extracellularly. It has been found that the yield and biochemical characteristics of the synthesized MTG vary significantly depending on the strain (Kieliszek and Misiewicz, 2014[15]; Akbari et al., 2021[1]). Work is still ongoing to isolate new strains of microorganisms capable of producing MTG with high efficiency from various environmental sources (Zhang et al., 2009[31]; Ceresino et al., 2018[3]). When evaluating the potential of new strains for enzyme production, it is crucial to adapt the appropriate nutrient composition for the cultivation of these specific strains (Ceresino et al., 2018[3]).

Despite the widespread use of transglutaminase in various industrial sectors (Akbari et al., 2021[1]), the *S. mobaraense* strain remains the only microorganism used for the commercial production of MTG. Unfortunately, there is a significant lack of knowledge regarding the production of transglutaminase by genetically unmodified strains of the *Streptoverticillium* genus, including *S. cinnamoneum*. Therefore, the results of this study should be compared to currently available publications on the production and activity of microbial transglutaminase from various strains of *Streptoverticillium*.

Most research results in the available literature are dedicated to the model representative of actinomycetes - *S. mobaraense*. In the study by Kieliszek and Misiewicz (2014[15]), the authors compiled the available knowledge on the activity of transglutaminase produced by *S. mobaraense* at that time. Results from the years 1994-2012 reported that scientists managed to determine MTG activity in the range of 0.9-3.4 U/mL. Other strains of the *Streptoverticillium* genus, such as *S. lividans*, *S. lydicus*, *S. platensis*, *S. sioyansis*, *S. griseocarneum*, exhibited MTG activity in the range of 1.3-2.2 U/mL.

In the study by Guerra-Rodríguez and Vázquez (2014[12]), which focused on the cost-optimal cultivation medium, transglutaminase activity was noted at 2.95 U/mL for *Streptomyces mobaraensis* CECT 3230. Two years later, Jin et al. (2016[13]) reported MTG activity of 1.75 U/mL for *Streptomyces mobaraensis* TX. In 2017, Xavier et al. (2017[29]), through nutrient optimization for *Streptomyces* sp., achieved transglutaminase activity at 4.1 U/mL, which was a record result in the literature on the subject. In 2018, Ceresino et al. (2018[3]) isolated a new strain, *Streptomyces* sp. CBMAI 1617 (SB6), which, in a substrate (composed of 2.5 % soybean meal, 2.0 % potato starch, 0.1 % glucose, 1.0 % bacteriological peptone, 0.4 % KH_2_PO_4_·7H_2_O and 0.2 % MgSO_4_·7H_2_O, pH 7.0), was capable of producing MTG with an activity of 6.07 U/mL.

The results of the current study provide new values for transglutaminase activity for the rarely described *S. cinnamoneum* strain. The studied strain exhibited MTG activity values ranging from 4.14 to 6.59 U/mL at various stages of the research. The highest result is 8.57 % higher than the result reported by Ceresino et al. (2018[3]), which is very promising for further research on this strain.

Another crucial aspect in studies on obtaining transglutaminase is the substrate composition, and more importantly, its costs, which can constitute almost 30 % of the overall expenses related to the biosynthesis process (Téllez-Luis et al., 2004[25]). In most of the studies concerning MTG biosynthesis from *Streptomyces* sp. strains, the nutrient compositions are nearly identical. Glucose and soluble starch are commonly used carbon sources, while aminobak, peptone and yeast extract serve as popular nitrogen sources in cultivation media for MTG production (Ando et al., 1989[2]; Akbari et al., 2021[1]). The substrates often contain essential elements such as sodium phosphate, potassium phosphate and magnesium sulphate (Guerra-Rodríguez and Vázquez, 2014[12]). It appears that supplementation with salts can effectively enhance MTG biosynthesis, probably by accelerating the transformation of pro-MTGase into a mature enzymatic domain through an increase in overall protease production (Fatima et al., 2019[10]). According to the results of Ceresino et al. (2018[3]), potassium dihydrogen phosphate is one of the components with the most positive and significant impact on transglutaminase biosynthesis, making it one of the ingredients used in this study.

Microbiological media used for cultivating *Streptoverticillium* strains are usually not economically viable owing to the high quantity of expensive nutritional components such as yeast extract and peptone (Kieliszek and Misiewicz, 2014[15]; Ceresino et al., 2018[3]). Many publications discuss the possibility of using agricultural waste as a source of carbon or nitrogen for transglutaminase production (Akbari et al., 2021[1]). Researchers Guerra-Rodríguez and Vázquez (2014[12]) evaluated MTG biosynthesis by *S. mobaraensis* using a substrate based on non-commercial potatoes. According to their results, the best medium turned out to be gelatinized, unhydrolyzed potato, enabling the attainment of enzyme activity up to 2.72 U/mL after 96 hours of cultivation. In another study conducted by Téllez-Luis et al. (2004[25]), the biosynthesis of transglutaminase by *Streptoverticillium ladakanum* NRRL-3191 was assessed using a medium containing a mixture of sorghum straw hydrolysate and xylose at a concentration of 20 g/L. Through these experiments, enzyme activity reached a level of 0.348 U/mL after 72 hours of cultivation. In a study by Portilla-Rivera et al. (2009[23]), the biosynthesis of MTG by *S. ladakanum* NRRL-3191 was evaluated on media prepared from molasses, sugar cane and glycerol. Results indicated that the highest MTGase activity (0.460 U/mL) was obtained in a medium containing a mixture of molasses and glycerol. In media containing only molasses from sugar cane and glycerol, enzyme activity was 0.240 U/mL and 0.250 U/mL, respectively.

In this study, an attempt was made to use corn steep liquor as a nitrogen source to replace the costly aminobak. Corn steep liquor, a product produced in the wet milling industry of corn, contains a high quantity of amino acids, polypeptides and B-group vitamins (Kim et al., 2020[16]). Studies have demonstrated that corn steep liquor is an excellent nitrogen source for most microorganisms (Yu et al., 2008[30]), especially those belonging to the *Streptomyces* genus (Nascimento et al., 2009[22]). By employing a research medium containing corn steep liquor and the investigated strain *S. cinnamoneum* KKP 1658, an MTG activity of 1.95 U/mL was achieved after 48 hours of cultivation. Compared to other waste components of substrates described above, the use of corn steep liquor translates into relatively high MTG activities.

Environmental conditions such as temperature and pH are crucial for effective MTG biosynthesis and must be optimized to enhance enzyme activity. However, various studies in this field often present conflicting results, which may arise from differences in microbial strains, media and other experimental factors. Here are a few examples from different studies: Zhang et al. (2012[32]) cultivated *S. mobaraensis* at a temperature of 30 °C and pH 7.0. Jin et al. (2016[13]) also cultivated *S. mobaraensis* at 30 °C but at pH 7.4. Turker et al. (2016[26]) determined that the optimal conditions for the highest enzyme activity were pH 6.0 and 30 °C for 14 days.

It appears that a temperature range of 28-30 °C and a pH around 7.0 are often favorable for achieving high MTG activity. However, fermentation time may vary depending on the cultivation conditions and the targeted enzyme activity, typically ranging from 72 to 96 hours. It is crucial to experimentally optimize these parameters in a specific case to achieve the best results in MTG biosynthesis (Kieliszek and Misiewicz, 2014[15]; Akbari et al., 2021[1]).

Due to conflicting literature results regarding the optimal cultivation conditions for *Streptoverticillium* strains and the lack of studies on the *S. cinnamoneum *KKP 1658 strain, it was decided to use a temperature of 28 °C and, based on the current research, select the initial pH of the medium within the range of 5.0-7.0. Summarizing the obtained results, it can be hypothesised that biomass obtained from a medium with an initial pH of 6.0 exhibits the highest transglutaminase activity.

It is essential to note that this study focuses on laboratory-scale research. A subsequent stage of research should be conducted, utilizing the previously presented results, by scaling up to bioreactor cultivations. Replicable results from bioreactor cultivations will provide detailed data on the average activity of microbial transglutaminase produced by the *Streptoverticillium cinnamoneum* KKP 1658 strain on a larger scale.

The results of this study provide evidence that the combination of commercial aminobak with waste corn steep liquor in a 1:1 ratio can yield higher transglutaminase activity than the individual nitrogen sources. Such a solution allows the highest MTG activity - up to 6.59 U/mL to be achieved while retaining economic benefits by replacing half of the expensive aminobak or peptone dose with waste corn steep liquor. Such a high MTG activity result may be associated with the composition of the waste material, which is rich in vitamins and amino acids, playing a crucial role in the microbial synthesis of transglutaminase.

## Conclusion

The investigated strain, *Streptoverticillium cinnamoneum* KKP 1658, cultivated in a medium containing aminobak and corn steep liquor as the primary nitrogen source, demonstrated the ability to biosynthesize transglutaminase with an activity of 6.59 U/mL. TSB proved to be a more effective inoculation medium than O_3_ with oat flakes. The optimal pre-inoculation time in the TSB inoculation medium was 24 hours, with an optimal inoculum dosage of 10 % of the experimental culture volume. Experimental cultures were conducted at 28 °C, and the initial pH in the range of 6.0-6.5 yielded the best transglutaminase activity results. It was observed that the highest biomass increase did not always correlate with the highest microbial transglutaminase activity. The use of corn steep liquor as a nitrogen source was justified, allowing for a 50 % reduction in costs associated with the purchase of expensive nitrogen sources such as aminobak or peptone. Furthermore, cultivation in this medium resulted in over a three-fold increase in transglutaminase activity compared to a medium with aminobak as the primary nitrogen source.

Currently, there is a lack of recent studies on the production of microbial transglutaminase by *S. cinnamoneum*, allowing the results of this study to fill the gap in the literature on this subject somewhat. In further stages of research, the focus should be on determining transglutaminase activity in bioreactor cultures, utilizing selected nitrogen sources as components of cultivation media.

## Declaration

### Acknowledgments

Research equipment was purchased as part of the “Food and Nutrition Centre-modernization of the WULS campus to create a Food and Nutrition Research and Development Centre (CŻiŻ)” project co-financed by the European Union from the European Regional Development Fund under the Regional Operational Programme of the Mazowieckie Voivodeship for 2014-2020 (Project No. RPMA.01.01.00-14-8276/17).

### Funding

Not applicable.

### Author contribution

Vitaliy Kolotylo: Methodology, data curation, investigation, writing - original draft, writing - review & editing. Kamil Piwowarek: Formal analysis, writing - review & editing. Marek Kieliszek: Conceptualization, methodology, data curation, investigation, writing - review & editing, supervision, project administration. All authors have read and agreed to the published version of the manuscript.

### Conflict of interest

Authors state no conflict of interest.

### Ethical approval

The conducted research is not related to either human or animal use.

### Data availability statement

The dataset generated during and/or analyzed during the current study are available from the first author on reasonable request. All data obtained were presented in the Supplementary data.

### Ethics approval 

This article does not involve human and animal research. The authors of this paper all participated in the work of the paper. All authors agree to participate in the writing of the paper and agree to publish this article. 

## Supplementary Material

Supplementary data
